# Editorial: Affective Sciences through the Chemical Senses

**DOI:** 10.3389/fpsyg.2016.01590

**Published:** 2016-10-19

**Authors:** Géraldine Coppin, Valentina Parma, Bettina M. Pause

**Affiliations:** ^1^Translational Neurocircuitry Group, Max Planck for Metabolism ResearchCologne, Germany; ^2^Swiss Center for Affective SciencesGeneva, Switzerland; ^3^Laboratory for the Study of Emotion Elicitation and Expression, Department of Psychology, University of GenevaGeneva, Switzerland; ^4^Neuroscience Area, International School for Advanced Studies (SISSA)Trieste, Italy; ^5^Monell Chemical Senses CenterPhiladelphia, PA, USA; ^6^Department of Clinical Neuroscience, Karolinska InstitutetStockholm, Sweden; ^7^Center for Autism Research, The Children's Hospital of PhiladelphiaPA, USA; ^8^Department of Experimental Psychology, Heinrich-Heine-University of DüsseldorfDüsseldorf, Germany

**Keywords:** olfaction, gustation, emotion, valence, affective neuroscience

Whence could it have come to me, this all-powerful joy? I was conscious that it was connected with the taste of tea and cake, but that it infinitely transcended those savors, could not, indeed, be of the same nature as theirs. Whence did it come? What did it signify? How could I seize upon and define it?

*Marcel Proust, À la Recherche du Temps Perdu (Proust, 1913–1927)*.

As the Proustian madeleine anecdote so well exemplifies, smells, and flavors can play a key role in mediating affective experiences. However, the intricacies of the human affective world, encompassing autonomic, sensory, motor, cognitive, and emotional aspects (e.g., Coppin and Sander, 2016), have to date left the link between chemosensory and affective sciences rather unexplored (e.g., Coppin et al., 2010). This Research Topic represents the efforts of two research fields to converge and explore the breath of their intersecting topics through the theoretical and experimental approaches of some of the researchers currently animating the field. The present E-book, therefore, offers a snapshot of the unique role of the chemical senses in shaping human affective experiences and vice-versa, i.e., in revealing how affective states influence chemosensation.

The prominent role of valence, the designation of positive and negative affects, in framing chemosensory experiences (e.g., Delplanque et al., 2016) made this topic the focus of several investigations included in the present Research Topic. This line of research origins from the idea that odors are powerful modulators of emotional experiences (e.g., Pause et al., 2003; Adolph and Pause, 2012). Delplanque et al. revealed that the so-called mere exposure effect depends on the initial pleasantness attributed to the odors. Indeed, the pleasantness to neutral and mildly pleasant olfactory stimuli increases following repeated exposure whereas it only marginally does that for overtly unpleasant and pleasant stimuli. These findings open an interesting speculation on the potential dangers of getting to like odors that are unpleasant and perhaps toxic, simply because they are regularly presented. The dangers of unpleasant/dangerous odors have been investigated by Wisman and Shrira, who studied participants' reactions to conscious and non-conscious exposure to putrescine, a chemical produced in the decaying tissues of dead bodies. In a set of four studies they showed, that putrescine can elicit threat management mechanisms, ranging from increased vigilance, to avoidance, and increased implicit cognitions toward escape. The importance of the valence is again emphasized by Hoenen et al. They investigated the mood effects following exposure to different smells. Although, the exposure to the odors—including the citrus odor, commonly claimed to have uplifting effects—did not prevent the negative mood to sink in, what maintained the happiness judgments elevated was the pleasantness judgment of the odor. Additionally, Pichon et al. provided evidence on the tools to evaluate subtle differences in emotional reactions to families of odors, subserving different functions. More specifically, they demonstrated that among series of odors varying in pleasantness, physiological reactions can differentiate odors from different families (e.g., fruity, animal), but not within the same family of fine fragrances.

Another set of contributions investigated affective chemosensation in the context of socio-emotional communication. Human body odors are stimuli producing important interpersonal information, able to promote adaptive effects on cognitive–affective processes (Semin and De Groot, 2013; Parma et al., 2016; Pause, 2016). One such example refers to the communication of categorical information such as gender. The work by Mutic et al. revealed that axillary odors were perceived as masculine, irrespective of the donor's effective gender; whereas a femininity bias is introduced by chemosignals during social perception. This study emphasized the need of considering gender effects in chemosensory perception and to further research the role chemosensory communication of sex and gender information play in social perception. An example of communication of transient information is provided by Wudarczyk et al. who evaluated the neurophysiological effects of chemosignals of anxiety during a Cyberball task, a socially threatening situation. Brain activations in areas linked to social rejection were blunted while smelling the anxiety body odors, suggesting a moderation effect on the social experience of exclusion. Besides the exclusive presentation of body odors, two studies investigated the interactions between body odors and fragrances. Allen et al. investigated the complementarity between fragrances and body odors and provided an indication that the choice of different fragrances is influenced by one's body odor features. In line with this evidence, Sorokowska et al. showed that body odors are relevant cues used to gather first impression judgments of certain personality traits, which can be modulated by the use of fragranced cosmetics over the natural body odor.

The previous authors have focused on the characterization of the intersection between chemosensory and affective sciences in healthy young adults. Five contributions have additionally investigated these interactions across the development as well as in special populations. Nováková et al. showed that the knowledge regarding the odors presented is critical in the chemosensory affective experience, even at a young age. In children 8–11 years old, the pleasantness of odors was modulated by the knowledge of their identity due to prior experience, but this effect was only confined to unpleasant odors. Ferdenzi et al. examined sniffing patterns in young and older healthy adults and showed that this behavior is sensitive to subtle variations in unpleasantness, even though in the elderly with lesser extent. Therefore, sniffing may have an adaptive function to protect individuals across the lifespan from inhaling harmful substances. Aging, among other causes, is associated with a reduction of olfactory ability, which can result in hyposmia or anosmia. Kollndorfer et al. investigated such populations with respect to the difference between objective (Sniffin' Sticks) and subjective olfactory performance (odor imagery) evaluation. They found a close relationship between the vividness of mental images and self-evaluation of olfactory perception abilities, suggesting that individuals subjectively did rate their olfactory performance, even when we are not able to perceive odors. To evaluate the neural underpinnings of affective chemosensory perception, Juran et al. tested patients with unilateral resection of the anterior medial temporal lobe with several olfactory tasks. Results indicated a keen role of the temporal lobe in odor identification, and of the left anterior temporal lobe in determining the emotional saliency to odors. Last, Luisier et al. compared odor perception between children with autism spectrum disorder and typically developing children. Critically, they showed that odor hedonic reactivity relates to food neophobia in children with autism spectrum disorder, which opens fascinating avenues of research at the intersection between autism, chemical senses and food intake research.

Overall, the variety of topics, techniques and populations included in the present Research Topic can be seen as proof of the widespread effects that the chemical senses play in affective sciences and vice-versa. We thank the authors for their contributions, which have highlighted the potential boundaries of this integrated field and its richness. With this Research Topic, we hold a promise toward a deeper understanding of the interactions between chemical senses and affective sciences from a psychological and neuroscientific perspective. This investigation will bring a mutual enrichment to both chemosensory and affective sciences, which—we hope—will continue to flourish in the future.

## Author contributions

GC, VP, and BP wrote the manuscript.

### Conflict of interest statement

The authors declare that the research was conducted in the absence of any commercial or financial relationships that could be construed as a potential conflict of interest.
